# Supplementary material for: Silibinin Suppresses the Hyperlipidemic Effects of the ALK-Tyrosine Kinase Inhibitor Lorlatinib in Hepatic Cells
Source: Int J Mol Sci. 2022 Sep 1;23(17):9986. doi: 10.3390/ijms23179986 (PMC9456400; doi:10.3390/ijms23179986)
Supplement: Supplementary file 1 [file ijms-23-09986-s001.zip › ijms-1877692-supplementary.pdf]

Silibinin suppresses the hyperlipidemic effects of the ALK-tyrosine kinase inhibitor lorlatinib in hepatic cells

SUPPLEMENTARY INFORMATION

(Figures S1 and S2, Tables S1 to S7)

A

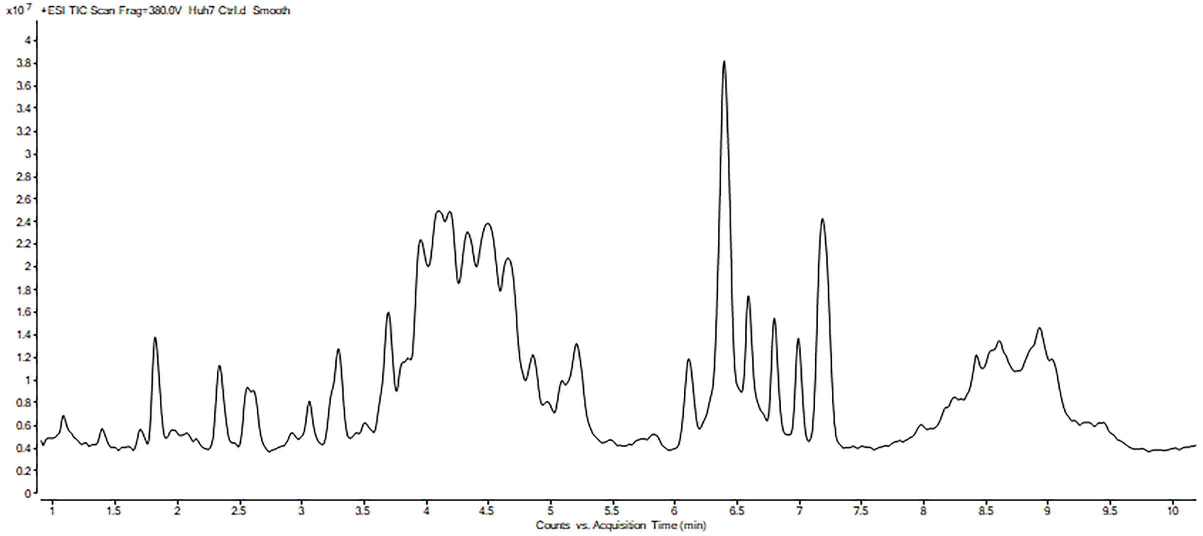

x10 7
+ESI TIC Scan Frag=380.0V Huh7 Ctrl.d Smooth
Counts vs. Acquisition Time (min)

B

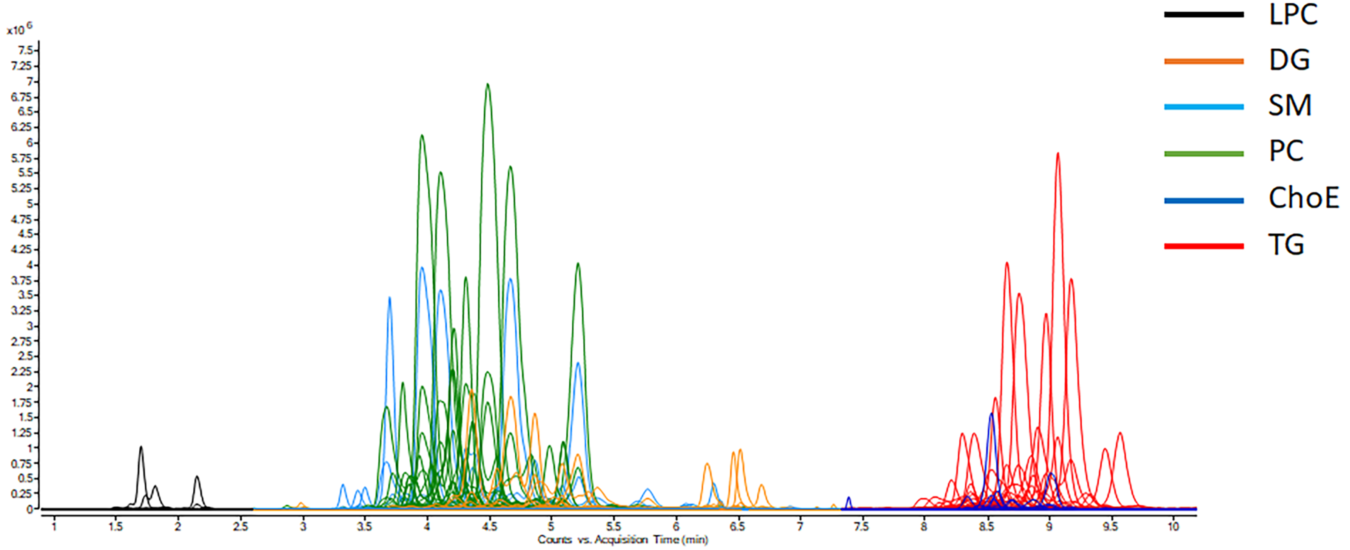

x10 6
LPC
DG
SM
PC
ChoE
TG
Counts vs. Acquisition Time (min)

Figure S1. Representative chromatograms of lipidomic analyses in Huh-7 cells. A. Total Ion Chromatogram (TIC) of lipidomic analysis using UHPLC-ESI-QTOF-MS/MS in untreated Huh-7 cells. **B.** Overlapped Extracted Ion Chromatograms (EIC) of different lipid species. ChoE: cholesteryl esters; DG: diglycerides; LCP: lysophosphatidylcholines; PC: phosphatidylcholines; SM: sphingomyelins; TG: triglycerides.

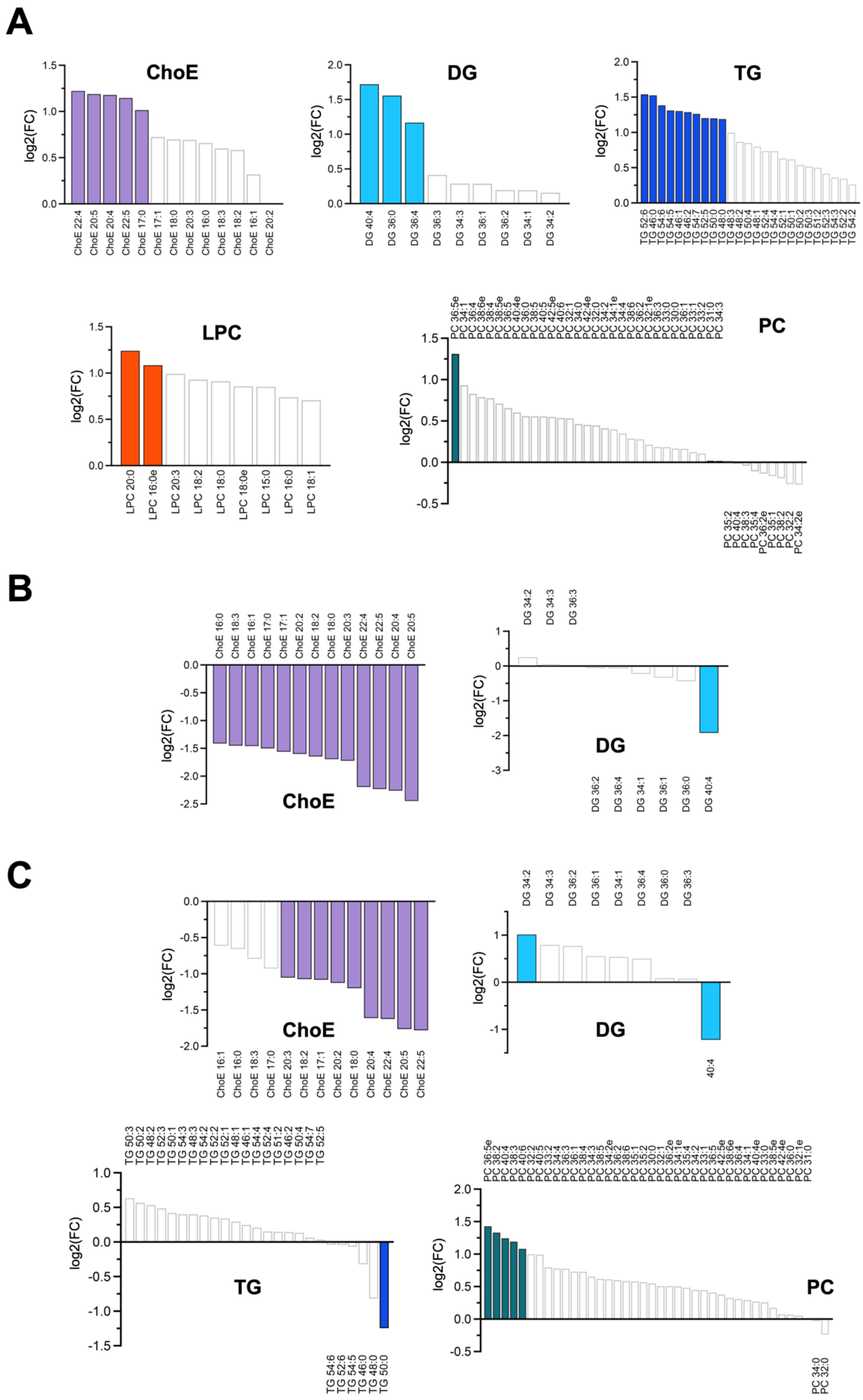

A
ChoE
log2(FC)
ChoE 22:4
ChoE 20:5
ChoE 20:4
ChoE 22:5
ChoE 17:0
ChoE 17:1
ChoE 18:0
ChoE 20:3
ChoE 16:0
ChoE 18:3
ChoE 18:2
ChoE 16:1
ChoE 20:2
DG
DG 40:4
DG 36:0
DG 36:4
DG 36:3
DG 34:3
DG 36:1
DG 36:2
DG 34:1
DG 34:2
TG
LPC
LPC 20:0
LPC 16:0e
LPC 20:3
LPC 18:2
LPC 18:0
LPC 18:0e
LPC 15:0
LPC 16:0
LPC 18:1
PC
B
C

Figure S2. Silibinin impedes lorlatinib-induced hypertriglyceridemia and hypercholesteremia traits in human hepatic cells. Fold-change (treatment/untreated control) of lipid species (expressed as median) in Huh-7 hepatocytes treated with 1 μmol/L lorlatinib (A), 100 μmol/L silibinin (B), and lorlatinib *plus* silibinin (C) for 48 h. Colored bars indicate statistically-significant ($p<0.05$) fold-changes. ChoE: cholesteryl esters; DG: diglycerides; TG: triglycerides; LPC: lysophosphatidylcholines; PC: phosphatidylcholines.

Table S1. Relative concentration (μmol/mg protein) of diglycerides (DG) in Huh-7 cells cultured (24 h) in the absence/presence of silibinin (SBN) and/or lorlatinib

	Untreated		SBN (100 μmol/L)		lorlatinib (1 μmol/L)		lorlatinib + SBN	
	**Median**	**(Q1–Q3)**	**Median**	**(Q1–Q3)**	**Median**	**(Q1–Q3)**	**Median**	**(Q1–Q3)**
**DG 34:1**	15.107	(12.395–16.562)	12.375	(10.746–14.38)	16.654	(15.513–17.831)	21.171	(18.457–23.958)
**DG 34:2**	16.979	(13.604–19.155)	19.08	(18.167–21.445)	17.722	(16.953–20.108)	32.692	(30.842–36.391)
**DG 34:3**	2.076	(1.724–2.430)	2.071	(1.983–2.351)	2.474	(2.244–2.845)	3.492	(3.319–3.964)
**DG 36:0**	0.774	(0.742–0.801)	0.566	(0.548–0.601)	2.250	(2.172– 2.375)	0.804	(0.786–0.865)
**DG 36:1**	8.068	(6.428–8.305)	5.747	(5.473–6.556)	8.885	(8.455–9.963)	10.95	(10.013–12.085)
**DG 36:2**	19.235	(15.459–20.836)	17.291	(16.302–19.224)	20.022	(19.455–22.991)	30.847	(29.449–33.641)
**DG 36:3**	1.540	(1.489–1.978)	1.684	(1.576–1.866)	2.186	(1.864–2.675)	1.682	(1.442–2.223)
**DG 36:4**	1.920	(1.734–1.994)	1.754	(1.680–1.934)	4.172	(3.809–4.624)	2.688	(2.437–2.825)
**DG 40:4**	2.844	(2.629–2.985)	0.721	(0.676–0.819)	9.158	(8.900–9.689)	1.209	(1.113–1.299)

Table S2. Relative concentration (μmol/mg protein) of triglycerides (TG) in Huh-7 cells cultured (24 h) in the absence/presence of silibinin (SBN) and/or lorlatinib

	Untreated		SBN (100 μmol/L)		lorlatinib (1 μmol/L)		lorlatinib + SBN	
	**Median**	**(Q1–Q3)**	**Median**	**(Q1–Q3)**	**Median**	**(Q1–Q3)**	**Median**	**(Q1–Q3)**
**TG 46:0**	0.626	(0.531–0.630)	0.522	(0.501–0.538)	1.549	(1.455–2.016)	0.462	(0.435–0.522)
**TG 46:1**	1.028	(0.890–1.077)	1.001	(0.959–1.059)	2.111	(1.967–3.099)	1.152	(1.021–1.356)
**TG 46:2**	0.468	(0.418–0.513)	0.491	(0.480–0.521)	1.025	(0.951–1.382)	0.504	(0.447–0.593)
**TG 48:0**	1.097	(1.060–1.188)	1.056	(0.882–1.112)	2.372	(2.147–3.040)	0.589	(0.431–0.886)
**TG 48:1**	3.172	(3.117–3.605)	3.468	(3.404–3.762)	5.318	(5.041–6.738)	4.132	(3.646–4.440)
**TG 48:2**	2.674	(2.574–2.848)	3.070	(2.971–3.286)	4.451	(4.275–5.807)	3.880	(3.292–4.554)
**TG 48:3**	0.629	(0.615–0.665)	0.647	(0.633–0.706)	1.146	(1.080–1.524)	0.809	(0.729–0.985)
**TG 50:0**	0.536	(0.504–0.557)	0.439	(0.360–0.484)	1.142	(1.089–1.393)	0.191	(0.139–0.342)
**TG 50:1**	6.317	(5.626–6.914)	6.316	(5.877–6.549)	9.399	(8.740–10.609)	8.418	(8.130–8.613)
**TG 50:2**	8.537	(7.504–9.159)	9.143	(8.771–9.523)	11.302	(11.039–13.647)	12.471	(12.143–12.484)
**TG 50:3**	3.380	(2.933–3.543)	3.680	(3.457–3.945)	4.462	(4.089–5.345)	5.041	(4.317–5.828)
**TG 50:4**	0.580	(0.552–0.603)	0.499	(0.468–0.529)	1.054	(0.880–1.195)	0.621	(0.529–0.751)
**TG 51:2**	2.259	(2.081–2.528)	1.903	(1.899–2.137)	2.969	(2.862–3.759)	2.621	(2.317–2.683)
**TG 52:1**	2.267	(2.003–2.420)	2.158	(1.870–2.248)	3.455	(2.862–4.004)	2.785	(2.723–2.920)
**TG 52:2**	12.662	(10.864–13.699)	11.698	(10.929–11.869)	16.188	(13.647–17.389)	15.768	(15.151–16.392)
**TG 52:3**	8.818	(7.839–9.243)	9.529	(8.596–9.700)	12.354	(10.026–12.385)	12.122	(11.482–12.493)
**TG 52:4**	2.087	(1.957–2.377)	1.894	(1.796–1.957)	3.787	(3.100–3.948)	2.399	(2.257–2.507)
**TG 52:5**	0.533	(0.521–0.553)	0.398	(0.387–0.418)	1.312	(1.062–1.370)	0.543	(0.471–0.633)
**TG 52:6**	0.246	(0.220–0.268)	0.183	(0.167–0.185)	0.708	(0.595–0.825)	0.232	(0.199–0.282)
**TG 54:2**	2.935	(2.514–3.326)	2.821	(2.359–2.944)	3.515	(3.239–3.759)	3.807	(3.545–4.069)
**TG 54:3**	9.029	(6.792–9.845)	7.594	(7.456–8.066)	11.096	(9.734–11.86)	11.245	(10.702–11.544)
**TG 54:4**	3.980	(3.492–4.291)	3.347	(3.084–3.473)	6.556	(5.885–7.076)	4.621	(4.101–4.792)
**TG 54:5**	1.686	(1.642–1.724)	1.234	(1.186–1.258)	4.411	(3.406–4.830)	1.609	(1.593–1.627)

**TG 54:6**	0.997	(0.960–1.142)	0.735	(0.683–0.746)	2.731	(2.242–3.179)	1.000	(0.960–1.076)
**TG 54:7**	0.931	(0.915–1.021)	0.693	(0.654–0.718)	2.095	(2.035–2.682)	0.982	(0.920–1.106)

Table S3. Relative concentration (μmol/mg protein) of phosphatidylcholines (PC) in Huh-7 cells cultured (24 h) in the absence/presence of silibinin (SBN) and/or lorlatinib

	Untreated		SBN (100 μmol/L)		lorlatinib (1 μmol/L)		lorlatinib + SBN	
	**Median**	**(Q1–Q3)**	**Median**	**(Q1–Q3)**	**Median**	**(Q1–Q3)**	**Median**	**(Q1–Q3)**
**PC 30:0**	6.405	(5.118–6.937)	5.379	(5.174–6.243)	6.654	(6.326–7.452)	9.079	(7.901–9.773)
**PC 31:0**	3.415	(2.727–3.774)	2.265	(2.157–2.574)	3.243	(3.017–3.670)	3.322	(3.059–3.496)
**PC 32:0**	15.784	(13.155–16.908)	9.342	(9.223–10.114)	20.672	(18.773–22.556)	12.665	(12.273–13.717)
**PC 32:1**	41.601	(34.358–45.369)	42.745	(41.334–46.236)	56.675	(54.510–62.270)	56.336	(54.025–60.809)
**PC 32:1e**	0.631	(0.549–0.745)	0.423	(0.381–0.652)	0.780	(0.662–0.811)	0.623	(0.438–0.941)
**PC 32:2**	10.379	(7.446–11.559)	12.917	(11.945–14.888)	7.800	(7.438–8.849)	19.704	(17.130–21.125)
**PC 33:0**	2.818	(2.327–3.265)	2.350	(2.268–2.621)	3.109	(2.967–3.427)	3.364	(3.092–3.573)
**PC 33:1**	12.16	(9.664–13.345)	11.141	(10.634–12.379)	12.338	(11.522–13.927)	15.984	(14.539–16.894)
**PC 33:2**	1.115	(0.854–1.266)	1.079	(1.007–1.384)	1.113	(1.064–1.257)	1.862	(1.634–2.076)
**PC 34:0**	4.608	(3.847–4.698)	2.771	(2.547–3.203)	5.917	(5.429–6.549)	4.239	(3.973–4.558)
**PC 34:1**	51.720	(43.818–55.688)	44.476	(43.811–48.347)	93.696	(91.390–100.415)	61.235	(57.209–65.474)
**PC 34:1e**	0.700	(0.520–0.787)	0.604	(0.568–0.715)	0.856	(0.799–0.950)	0.935	(0.851–1.028)
**PC 34:2**	38.098	(31.369–41.923)	37.539	(36.238–40.854)	49.219	(44.698–53.364)	50.38	(47.057–53.648)
**PC 34:2e**	0.874	(0.713–0.998)	0.672	(0.640–0.907)	0.709	(0.675–0.755)	1.408	(1.019–1.506)
**PC 34:3**	5.841	(4.462–6.531)	5.903	(5.612–6.805)	5.578	(5.118–6.157)	8.815	(7.869–9.525)
**PC 34:4**	0.771	(0.614–0.836)	0.831	(0.780–0.967)	0.930	(0.844–1.024)	1.286	(1.120–1.367)
**PC 35:1**	7.539	(5.623–8.474)	6.545	(6.327–7.730)	6.392	(5.728–7.026)	10.867	(9.444–11.642)
**PC 35:2**	6.528	(5.154–7.323)	6.508	(6.177–7.348)	6.219	(5.750–6.996)	9.368	(8.563–10.009)
**PC 35:4**	1.991	(1.639–2.367)	2.145	(2.036–2.373)	1.817	(1.637–2.103)	2.919	(2.289–3.166)
**PC 36:0**	0.144	(0.136–0.154)	0.086	(0.085–0.107)	0.209	(0.187–0.240)	0.156	(0.126–0.172)
**PC 36:1**	23.236	(18.201–25.375)	23.301	(22.443–26.714)	24.222	(22.536–27.057)	36.729	(32.578–40.415)
**PC 36:2**	40.944	(32.394–45.118)	39.690	(39.461–44.726)	46.840	(44.353–50.893)	59.556	(54.484–63.853)
**PC 36:2e**	0.114	(0.083–0.131)	0.100	(0.095–0.106)	0.101	(0.090–0.106)	0.154	(0.127–0.180)

**PC 36:3**	19.137	(15.120–21.227)	21.699	(21.136–25.041)	20.945	(18.943–22.717)	31.596	(28.917–33.683)
**PC 36:4**	14.654	(13.650–15.620)	13.768	(12.888–14.909)	26.422	(22.340–29.315)	18.568	(16.184–19.618)
**PC 36:5**	4.383	(4.014–4.479)	4.055	(3.684–4.522)	6.790	(6.113–7.305)	5.650	(5.331–6.025)
**PC 36:5e**	0.304	(0.265–0.553)	0.838	(0.531–0.900)	0.749	(0.597–1.436)	1.189	(0.509–1.437)
**PC 38:2**	2.035	(1.630–2.307)	3.083	(2.704–3.763)	1.747	(1.526–1.942)	4.971	(4.247–5.735)
**PC 38:3**	3.083	(2.572–3.732)	4.548	(4.312–5.591)	2.875	(2.711–3.493)	7.412	(6.261–7.813)
**PC 38:4**	6.837	(6.203–7.052)	7.055	(6.730–8.292)	12.355	(8.634–13.664)	10.930	(10.219–11.943)
**PC 38:5**	9.205	(6.367–9.755)	7.047	(6.709–8.775)	12.838	(10.587–13.421)	12.874	(11.692–13.573)
**PC 38:5e**	0.124	(0.112–0.130)	0.125	(0.048–0.126)	0.193	(0.176–0.225)	0.132	(0.099–0.179)
**PC 38:6**	7.724	(6.355–8.296)	7.943	(7.652–8.893)	9.312	(7.567–10.337)	11.184	(9.903–12.163)
**PC 38:6e**	0.222	(0.200–0.258)	0.201	(0.128–0.223)	0.353	(0.316–0.487)	0.299	(0.221–0.33)
**PC 40:4**	0.312	(0.232–0.360)	0.457	(0.425–0.558)	0.298	(0.264–0.327)	0.701	(0.608–0.813)
**PC 40:4e**	0.064	(0.054–0.068)	0.050	(0.045–0.056)	0.087	(0.085–0.105)	0.076	(0.058–0.089)
**PC 40:5**	1.333	(1.178–1.438)	1.568	(1.550–1.921)	1.883	(1.759–2.114)	2.582	(2.315–2.926)
**PC 40:6**	2.004	(1.843–2.221)	2.507	(2.286–3.134)	2.981	(2.661–3.179)	4.222	(3.863–4.769)
**PC 42:4e**	0.042	(0.036–0.046)	0.034	(0.031–0.040)	0.056	(0.05–0.063)	0.043	(0.04–0.046)
**PC 42:5e**	0.084	(0.067–0.104)	0.085	(0.079–0.098)	0.118	(0.114–0.139)	0.117	(0.09–0.126)

Table S4. Relative concentration (μmol/mg protein) of lysophosphatidylcholines (LPC) in Huh-7 cells cultured (24 h) in the absence/presence of silibinin (SBN) and/or lorlatinib

	Untreated		SBN (100 μmol/L)		lorlatinib (1 μmol/L)		lorlatinib + SBN	
	**Median**	**(Q1–Q3)**	**Median**	**(Q1–Q3)**	**Median**	**(Q1–Q3)**	**Median**	**(Q1–Q3)**
**LPC 15:0**	0.052	(0.041–0.067)	0.045	(0.037–0.062)	0.094	(0.082–0.112)	0.056	(0.047–0.083)
**LPC 16:0**	2.004	(1.575–2.348)	2.095	(1.794–2.953)	3.165	(2.917–3.708)	2.901	(2.366–3.731)
**LPC 16: e**	0.012	(0.009–0.014)	0.007	(0.006–0.009)	0.023	(0.022–0.027)	0.009	(0.008–0.011)
**LPC 18:0**	1.105	(0.885–1.223)	1.353	(1.132–1.761)	1.911	(1.805–2.236)	1.862	(1.555–2.259)
**LPC 18:0e**	0.008	(0.007–0.008)	0.006	(0.005–0.007)	0.014	(0.012–0.015)	0.007	(0.006–0.009)
**LPC 18:1**	1.065	(0.731–1.434)	0.842	(0.721–1.339)	1.630	(1.184–2.396)	1.163	(0.973–1.883)
**LPC 18:2**	0.106	(0.063–0.143)	0.068	(0.060–0.123)	0.188	(0.118–0.282)	0.096	(0.079–0.179)
**LPC 20:0**	0.022	(0.021–0.023)	0.029	(0.024–0.03)	0.047	(0.046–0.059)	0.039	(0.031–0.041)
**LPC 20:3**	0.058	(0.028–0.075)	0.030	(0.024–0.06)	0.080	(0.049–0.173)	0.044	(0.036–0.096)

Table S5. Relative concentration (μmol/mg protein) of phosphatidylethanolamines (PE) in Huh-7 cells cultured (24 h) in the absence/presence of silibinin (SBN) and/or lorlatinib

	Untreated		SBN (100 μmol/L)		lorlatinib (1 μmol/L)		lorlatinib + SBN	
	**Median**	**(Q1–Q3)**	**Median**	**(Q1–Q3)**	**Median**	**(Q1–Q3)**	**Median**	**(Q1–Q3)**
**PE 32:0**	0.121	(0.108–0.141)	0.115	(0.086–0.125)	0.231	(0.201–0.271)	0.137	(0.129–0.178)
**PE 36:4**	12.901	(8.92–13.929)	14.38	(13.745–16.186)	15.311	(14.832–17.361)	18.76	(17.928–19.9375)
**PE 36:5e**	0.714	(0.665–0.779)	0.673	(0.569–0.733)	1.339	(0.925–1.395)	1.026	(0.954–1.0558)
**PE 38:5e**	0.381	(0.322–0.393)	0.321	(0.302–0.386)	0.649	(0.592–0.743)	0.491	(0.461–0.514)

Table S6. Relative concentration (μmol/mg protein) of sphingomyelins (SM) in Huh-7 cells cultured (24 h) in the absence/presence of silibinin (SBN) and/or lorlatinib

	Untreated		SBN (100 μmol/L)		lorlatinib (1 μmol/L)		lorlatinib + SBN	
	Median	(Q1–Q3)	Median	(Q1–Q3)	Median	(Q1–Q3)	Median	(Q1–Q3)
**SM 32:0**	0.296	(0.240–0.334)	0.408	(0.392–0.450)	0.350	(0.343–0.395)	0.539	(0.508–0.569)
**SM 32:1**	1.361	(1.167–1.538)	1.232	(1.186–1.395)	2.087	(1.758–2.338)	1.810	(1.645–2.113)
**SM 32:2**	0.021	(0.016–0.022)	0.016	(0.013–0.017)	0.032	(0.024–0.032)	0.020	(0.019–0.022)
**SM 33:1**	1.395	(1.174–1.549)	1.184	(1.119–1.307)	1.877	(1.798–2.085)	1.618	(1.505–1.748)
**SM 34:1**	16.065	(13.679–17.477)	13.074	(12.542–13.614)	22.397	(20.913–24.645)	16.438	(15.900–17.524)
**SM 34:2**	1.406	(1.185–1.494)	1.186	(1.157–1.323)	1.973	(1.880–2.189)	1.677	(1.572–1.773)
**SM 35:0**	1.800	(1.363–1.958)	1.888	(1.764–2.167)	1.702	(1.617–1.855)	2.674	(2.416–2.881)
**SM 35:1**	0.611	(0.513–0.678)	0.369	(0.355–0.400)	0.803	(0.742–0.888)	0.486	(0.462–0.517)
**SM 36:0**	0.177	(0.156–0.186)	0.128	(0.126–0.132)	0.297	(0.284–0.335)	0.143	(0.132–0.150)
**SM 36:1**	0.938	(0.873–0.997)	0.555	(0.532–0.561)	1.764	(1.596–1.956)	0.614	(0.601–0.643)
**SM 36:2**	0.491	(0.420–0.530)	0.390	(0.375–0.427)	0.702	(0.633–0.757)	0.530	(0.503–0.558)
**SM 38:1**	0.421	(0.371–0.466)	0.310	(0.303–0.345)	0.595	(0.574–0.639)	0.412	(0.379–0.444)
**SM 38:2**	0.081	(0.075–0.085)	0.054	(0.049–0.054)	0.151	(0.145–0.171)	0.055	(0.054–0.058)
**SM 39:1**	0.142	(0.121–0.153)	0.107	(0.104–0.121)	0.210	(0.198–0.234)	0.136	(0.123–0.146)
**SM 40:0**	0.177	(0.126–0.205)	0.206	(0.203–0.232)	0.286	(0.274–0.326)	0.269	(0.231–0.289)
**SM 40:1**	1.146	(0.937–1.220)	0.875	(0.795–0.943)	1.575	(1.498–1.852)	1.169	(1.022–1.248)
**SM 40:2**	0.678	(0.568–0.720)	0.520	(0.500–0.558)	0.891	(0.785–0.983)	0.670	(0.628–0.726)
**SM 41:1**	0.487	(0.395–0.522)	0.392	(0.351–0.423)	0.637	(0.579–0.762)	0.554	(0.527–0.598)
**SM 41:2**	0.034	(0.031–0.044)	0.031	(0.026–0.032)	0.065	(0.054–0.078)	0.041	(0.036–0.046)
**SM 42:1**	1.434	(1.288–1.745)	1.615	(1.513–1.713)	2.064	(1.676–2.242)	1.855	(1.698–1.918)
**SM 42:2**	2.493	(2.035–2.733)	2.190	(2.075–2.375)	3.149	(2.980–3.542)	2.946	(2.616–3.172)

**SM 42:3**	0.630	(0.524–0.655)	0.411	(0.395–0.413)	0.805	(0.789–0.950)	0.420	(0.388–0.486)
**SM 43:1**	0.104	(0.087–0.130)	0.089	(0.087–0.105)	0.128	(0.119–0.146)	0.091	(0.089–0.097)
**SM 43:2**	0.301	(0.241–0.337)	0.232	(0.224–0.255)	0.358	(0.339–0.401)	0.309	(0.287–0.329)

Table S7. Relative concentration (μmol/mg protein) of cholesterol esters (ChoE) in Huh-7 cells cultured (24 h) in the absence/presence of silibinin (SBN) and/or lorlatinib

	Untreated		SBN (100 μmol/L)		lorlatinib (1 μmol/L)		Lorlatinib + SBN	
	Median	(Q1–Q3)	Median	(Q1–Q3)	Median	(Q1–Q3)	Median	(Q1– Q3)
**ChoE 16:0**	8.017	(6.695–8.384)	2.937	(2.566–3.137)	11.335	(11.192–13.242)	4.902	(4.288–5.361)
**ChoE 16:1**	18.886	(16.302–26.857)	7.298	(6.573–8.925)	25.820	(23.602–28.986)	13.832	(12.687–14.819)
**ChoE 17:0**	1.080	(0.970–1.130)	0.371	(0.367–0.382)	2.170	(2.030–2.215)	0.568	(0.493–0.607)
**ChoE 17:1**	3.697	(3.306–3.962)	1.214	(1.130–1.355)	5.670	(5.415–6.781)	1.771	(1.545–1.854)
**ChoE 18:0**	2.500	(2.398–2.926)	0.835	(0.752–0.865)	4.365	(3.681–4.782)	1.107	(1.064–1.269)
**ChoE 18:2**	23.460	(20.805–25.330)	7.393	(7.038–7.777)	33.284	(30.618–39.288)	11.057	(10.731–11.237)
**ChoE 18:3**	9.300	(8.319–9.577)	3.182	(3.139–3.520)	12.818	(12.373–15.356)	5.188	(4.825–5.634)
**ChoE 20:2**	6.224	(5.056–7.357)	2.088	(1.884–2.195)	6.459	(5.193–7.023)	2.892	(2.36–3.299)
**ChoE 20:3**	29.276	(22.698–30.894)	7.915	(7.695–9.037)	42.167	(41.084–48.000)	13.402	(12.338–13.755)
**ChoE 20:4**	51.820	(46.507–56.194)	10.818	(10.014–11.434)	107.967	(104.761–132.255)	16.964	(16.229–17.320)
**ChoE 20:5**	32.124	(29.012–37.344)	5.857	(5.696–6.506)	68.788	(65.756–87.599)	9.858	(9.128–10.151)
**ChoE 22:4**	12.329	(11.526–12.776)	2.650	(2.547–2.793)	27.538	(24.473–32.807)	4.093	(3.542–4.234)
**ChoE 22:5**	27.912	(26.452–30.841)	5.862	(5.682–6.574)	59.058	(55.8–72.323)	9.102	(6.283–9.537)
